# Mixed ductal‐lobular carcinomas: evidence for progression from ductal to lobular morphology

**DOI:** 10.1002/path.5040

**Published:** 2018-03-09

**Authors:** Amy E McCart Reed, Jamie R Kutasovic, Katia Nones, Jodi M Saunus, Leonard Da Silva, Felicity Newell, Stephen Kazakoff, Lewis Melville, Janani Jayanthan, Ana Cristina Vargas, Lynne E Reid, Jonathan Beesley, Xiao Qing Chen, Anne-Marie Patch, David Clouston, Alan Porter, Elizabeth Evans, John V Pearson, Georgia Chenevix‐Trench, Margaret C Cummings, Nicola Waddell, Sunil R Lakhani, Peter T Simpson

**Affiliations:** ^1^ Centre for Clinical Research, Faculty of Medicine The University of Queensland Brisbane Australia; ^2^ QIMR Berghofer Medical Research Institute Brisbane Australia; ^3^ Pathology Queensland The Royal Brisbane and Women's Hospital Brisbane Australia; ^4^ TissuPath, Mt Waverley Melbourne Australia; ^5^ The Wesley Breast Clinic The Wesley Hospital Brisbane Australia

**Keywords:** breast cancer, mixed ductal–lobular carcinoma, invasive lobular carcinoma, pathology, morphology, tumour heterogeneity, E‐cadherin, clonality

## Abstract

Mixed ductal–lobular carcinomas (MDLs) show both ductal and lobular morphology, and constitute an archetypal example of intratumoural morphological heterogeneity. The mechanisms underlying the coexistence of these different morphological entities are poorly understood, although theories include that these components either represent ‘collision’ of independent tumours or evolve from a common ancestor. We performed comprehensive clinicopathological analysis of a cohort of 82 MDLs, and found that: (1) MDLs more frequently coexist with ductal carcinoma in situ (DCIS) than with lobular carcinoma in situ (LCIS); (2) the E‐cadherin–catenin complex was normal in the ductal component in 77.6% of tumours; and (3) in the lobular component, E‐cadherin was almost always aberrantly located in the cytoplasm, in contrast to invasive lobular carcinoma (ILC), where E‐cadherin is typically absent. Comparative genomic hybridization and multiregion whole exome sequencing of four representative cases revealed that all morphologically distinct components within an individual case were clonally related. The mutations identified varied between cases; those associated with a common clonal ancestry included BRCA2, TBX3, and TP53, whereas those associated with clonal divergence included CDH1 and ESR1. Together, these data support a model in which separate morphological components of MDLs arise from a common ancestor, and lobular morphology can arise via a ductal pathway of tumour progression. In MDLs that present with LCIS and DCIS, the clonal divergence probably occurs early, and is frequently associated with complete loss of E‐cadherin expression, as in ILC, whereas, in the majority of MDLs, which present with DCIS but not LCIS, direct clonal divergence from the ductal to the lobular phenotype occurs late in tumour evolution, and is associated with aberrant expression of E‐cadherin. The mechanisms driving the phenotypic change may involve E‐cadherin–catenin complex deregulation, but are yet to be fully elucidated, as there is significant intertumoural heterogeneity, and each case may have a unique molecular mechanism. © 2018 The Authors. *The Journal of Pathology* published by John Wiley & Sons Ltd on behalf of Pathological Society of Great Britain and Ireland.

## Introduction

The full extent of breast cancer heterogeneity is being recognized through genomic analysis, although its visible manifestations have been essential to the histopathological classification for many decades. Breast tumours are broadly categorized into two morphological groups: tumours showing specialized growth patterns, and those with no distinguishing features [invasive carcinoma of no special type (IC‐NST), or invasive ductal carcinoma (IDC), which we will use for simplification of expression]. Invasive lobular carcinoma (ILC) is the most common special type (∼15%), and is defined by discohesive cells individually dispersed or arranged in single file, linearly in the fibrous stroma 1. Three to five per cent of tumours show both ductal and lobular morphology 1, and are classified as mixed ductal–lobular carcinoma (MDL) if the ductal component constitutes at least 10% of the tumour and the lobular component constitutes ≥50% 1, 2, 3. However, this definition is interpreted variably, as it does not account for tumours showing small foci of lobular differentiation. Some therefore report MDL as IDC with lobular features or simply as ‘ductal or lobular carcinoma’, whereas others also include those tumours with predominantly ductal features 3.

Several studies have investigated the clinical significance of MDLs, and have shown that they are associated with a better prognosis than IDC, but a poorer prognosis than ILC 3, 4, although the distinction was mostly lost after adjustment for grade 3, and that the prognosis for MDLs was poorer after stratification for oestrogen receptor (ER) positivity 5. Whereas a number of studies have shown that preinvasive lesions confined to the duct [lobular carcinoma *in situ* (LCIS) and ductal carcinoma *in situ* (DCIS)] and their associated invasive carcinomas are clonally related 6, 7, 8, there are insufficient studies to support this idea in MDLs. Limited evidence suggests that lesions with mixed morphology are clonally related and arise from a common ancestor 9; however, this study used low‐resolution technologies on relatively small cohorts. MDLs represent a unique model for interrogating intratumoural heterogeneity, clonal evolution, and the mechanisms driving acquisition of infiltrative growth patterns. In contrast to ILC and IDC, there are limited data on the underlying pathobiology of MDLs 2, 3.

To further understand intratumoural heterogeneity and clonal origins of the tumour, we assembled a cohort of 82 MDLs and investigated the expression of the E‐cadherin adhesion complex, which is lost in classic ILC, accounting for its characteristic morphology. We also performed detailed genomic analysis of representative MDLs, using chromosomal comparative genomic hybridization (cCGH) (*n* = 4) and whole exome sequencing (WES) (*n* = 4) to investigate clonality. We show that the lobular and ductal components within MDLs arise from a common ancestor, as opposed to the collision of two independent tumours, and that the phenotypic diversity is a result of clonal progression.

## Materials and methods

### Clinical cohort

Clinical and pathological data were obtained for a total of 82 patients, and samples were obtained for 51 patients (supplementary material, Table S1). Archival formalin‐fixed paraffin‐embedded (FFPE) tissue was obtained from local pathology laboratories, and frozen tissue and blood DNA were obtained from the Brisbane Breast Bank. Human research ethics committees approved the use of all clinical samples [University of Queensland (2005000785), Royal Brisbane and Women's Hospital (2005/022), and QIMR Berghofer (P2091)]. Multiple slides per case were reviewed, by several contributing authors at various stages of the project (L.D.S., A.C.V., L.M., D.C., M.C., P.T.S., and S.R.L.).

### Immunohistochemistry (IHC)

IHC for the E‐cadherin complex was performed (on whole tissue sections; antibodies and staining conditions are detailed in supplementary materials and methods) and scored centrally. E‐cadherin and its binding partners were scored as negative in the absence of expression, or positive when there was complete, linear membranous staining. Aberrant staining was defined as either fragmented membranous staining (i.e. non‐linear) or staining with a diffuse cytoplasmic localization. Positive/aberrant staining was recorded in those cases that showed both positive membranous staining and aberrant staining. The expression of ER, progesterone receptor (PR) and human epidermal growth factor receptor 2 (HER2) for each case was retrieved from pathology reports.

### Genomic analyses

Tumour‐rich regions were microdissected and DNA was extracted with the Qiagen (Melbourne, Australia) QIAamp DNA Micro Kit. cCGH was performed and the results were analysed as previously described 10, and WES was performed and the results were analysed as described in supplementary materials and methods.

## Results

### Unique clinical and pathological features of MDLs

The clinical and pathological features of 82 MDLs were compared with those of a cohort of 256 IDCs and 64 ILCs from the Queensland Follow‐Up (QFU) cohort 11, 12, 13, 14 (Table 1). The average age at MDL diagnosis was 57 years, which was significantly younger than that for ILC (62 years; *p* = 0.0083), but not that for IDC (58 years). The frequency of grade 2 MDL tumours was significantly higher than that of IDC tumours (*p* = 0.0254), but lower than that of ILC tumours (*p* = 0.0006). MDL patients presented more frequently with lymph node metastases than did IDC and ILC patients (*p* = 0.0033 and *p* = 0.0097, respectively).

**Table 1:** Clinical and pathological features of MDLs as compared with a sporadic breast cancer cohort

	MDL (*n* = 82)	IDC (*n* = 256)		ILC (*n* = 64)	
Clinicopathological feature	*n* (%)	*n* (%)	*P* value	*n* (%)	*P* value
Age at diagnosis (years)			0.3238^a^		0.0083^a^
Average	57	58		62	
Range	28–86	27–88		40–85	
Median	55	58		63	
Tumour size (cm)			0.2929		0.2017
<2	33 (42.9)	108 (42.2)		21 (34.4)	
2–5	33 (42.9)	92 (35.9)		24 (39.3)	
>5	11 (14.3)	56 (21.9)		16 (26.2)	
Not reported	5	–		3	
Total	82	256		64	
Tumour grade^b^			0.0254		0.0006
1	9 (11.0)	40 (15.6)		2 (3.1)	
2	48 (58.5)	106 (41.4)		56 (87.5)	
3	25 (30.5)	110 (43.0)		6 (9.4)	
Lymph node status			0.0033^b^		0.0097^b^
Positive	41 (68.3)	65 (41.1)		14 (40.0)	
Negative	19 (31.7)	79 (54.9)		21 (60.0)	
Not reported	22	112		29	
Total	82	256		64	
*In situ* lesions			<0.0001		<0.000
DCIS only	35 (60.3)	119 (100)		0 (0)	
LCIS only	7 (12.1)	0 (0)		29 (93.5)	
DCIS + LCIS	16 (27.6)	0		2 (6.5)	
Not reported	24	137		33	
ER			0.0102^b^		0.7842^b^
Positive	72 (90.0)	192 (76.8)		53 (91.4)	
Negative	8 (10.0)	58 (23.3)		5 (8.6)	
Not reported	2	6		6	
Total	82	256		64	
PR			0.0003^b^		0.0651^b^
Positive	67 (83.8)	154 (62.1)		38 (70.4)	
Negative	13 (16.3)	94 (37.9)		16 (29.6)	
Not reported	2	8		10	
Total	82	256		64	
HER2 (IHC)			1.0^b^		0.1255^b^
Positive	14 (18.2)	45 (18.7)		4 (8.0)	
Negative	63 (81.8)	196 (81.3)		46 (92.0)	
Not reported	5	15		14	
Total	82	256		64	
*HER2* (ISH)			0.2181^b^		0.0196^b^
Positive	8 (17.8)	27 (11.2)		2 (3.4)	
Negative	37 (82.2)	215 (88.8)		56 (96.6)	
Total	45	242		58	

ISH, *in situ* hybridization.

Chi‐square test unless indicated.

A *P* value of <0.05 is considered to be significant.

a *t*‐test.

b Fisher's exact test.

*In situ* carcinoma was diagnosed in 58 of 82 cases, with DCIS in 51 of 58 cases (87.9%). Of these, 35 (60.3%) presented with DCIS alone, 16 (27.6%) presented with DCIS coincident with LCIS, and just seven (12%) presented with LCIS only. The distribution of DCIS and LCIS in MDLs was significantly different from that in the QFU cohort (*p* < 0.0001; Table 1). For instance, there were significantly fewer cases with LCIS only in the MDL cohort than in the ILC cohort (*p* < 0.0001). Regarding hormone receptor expression, MDLs expressed ER and PR more frequently than IDCs (*p* = 0.0102 and *p* = 0.0003, respectively), but there was no difference in expression as compared with ILCs. None of the MDL tumours expressed basal markers [cytokeratin (CK) 5/6; CK14; and epidermal growth factor receptor].

### The E‐cadherin adhesion complex is aberrant but not lost in the lobular component of MDLs

**Figure 1:**
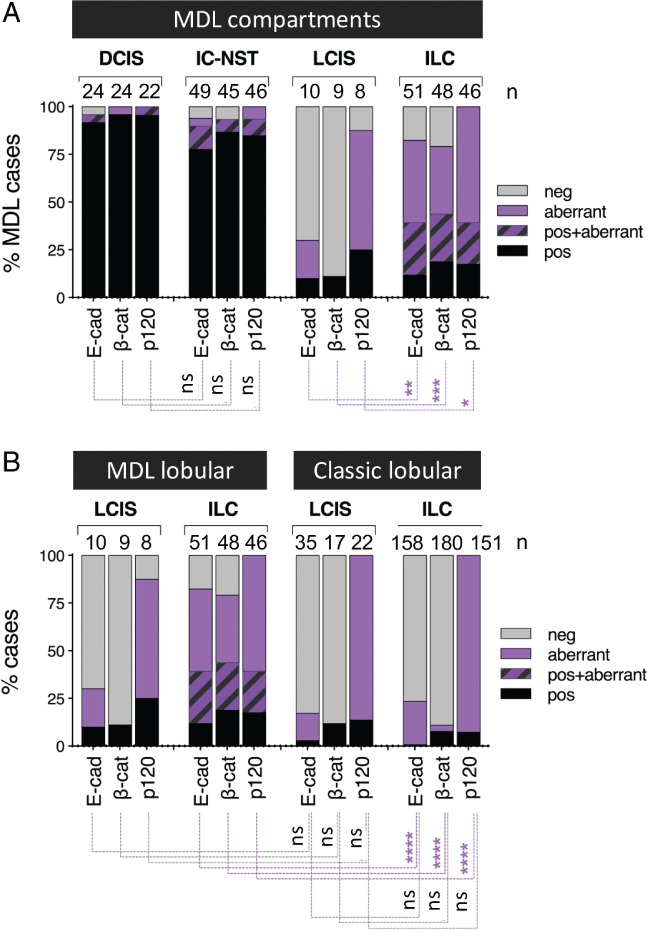
Expression of E‐cadherin complex proteins in MDLs. (A) In situ and invasive components of MDLs variably express E‐cadherin, β‐catenin, and p120 catenin. Unlike in LCIS, E‐cadherin and β‐catenin are aberrantly expressed as opposed to lost in the lobular components of MDLs. (B) The E‐cadherin adhesion complex shows similar expression patterns in LCIS lesions in both MDLs and genuine ILCs, whereas the expression of these proteins in the invasive lobular components of MDLs is significantly different from that seen in ILCs. Chi‐square analysis: ****p < 0.000000001; ***p = 0.0008; **p < 0.005; *p < 0.01. E‐cad, E‐cadherin; neg, negative; ns, not significant; pos, positive; β‐cat, β‐catenin.

A characteristic feature of the ILC phenotype is cellular discohesion as a result of E‐cadherin complex dysfunction 15, 16, and is classically observed as complete loss of E‐cadherin and β‐catenin staining, with relocalization of p120 catenin to the cytoplasm. The integrity of E‐cadherin and its binding partners was assessed (Figure 1). Both *in situ* and invasive components of ductal morphology typically showed normal, membranous staining for E‐cadherin, β‐catenin, and p120 catenin. The majority of LCISs were negative for E‐cadherin (70%) and β‐catenin (88.9%), with cytoplasmic p120 catenin (62.5%). However, in the invasive lobular compartment, just 17.6% of cases (9/51) had lost E‐cadherin expression, with most showing aberrant staining.

We compared patterns of E‐cadherin complex expression between lobular components of the MDLs with a cohort of 148 pure ILCs and associated LCISs 17. Both *in situ* and invasive components of pure ILCs showed loss of E‐cadherin and β‐catenin significantly more frequently than the lobular components of MDLs; conversely, the lobular components of MDLs showed aberrant expression of the complex significantly more frequently than pure ILCs (Figure 1B; *p* < 0.0001).

**Figure 2:**
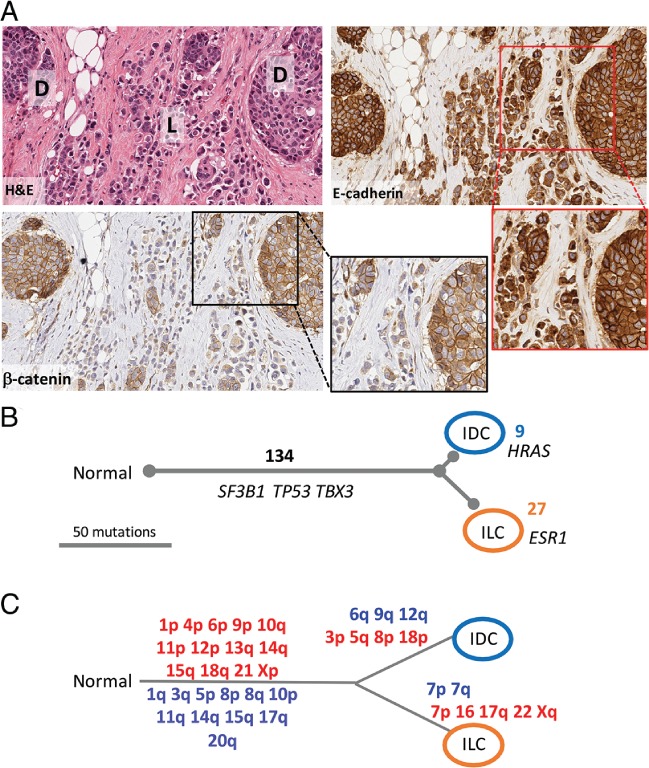
Detailed genomic evolution of morphologically distinct components of MDL4. (A) Morphology of MDL4. A haematoxylin and eosin (H&E)‐stained section shows the admixed relationship between tumour nests and discohesive single cells and single‐cell files. E‐cadherin and β‐catenin immunohistochemical staining demonstrates the clear phenotypic difference between the solid ductal component (D) and the discohesive lobular component (L), whereas the E‐cadherin staining in the lobular component remains strong, but is observed as cytoplasmic or discrete perinuclear dots, and β‐catenin staining is weak to negative. Note the insets showing high‐power fields. (B) Exome sequencing analysis of the invasive components identified a number of alterations; 134 variants were common to both lesions, including non‐synonymous changes in the breast cancer driver genes (albeit at low frequencies in the ductal component) SF3B1, TBX3, and TP53. Nine variants were unique to the ductal region, and included the cancer driver gene HRAS; 27 variants were unique to the lobular component, including ESR1. Variants included in this analysis were both synonymous and non‐synonymous, and also low‐frequency alleles not definitively called as ‘mutations’ by the analysis pipeline. Numbers in black represent shared variants along ‘trunks’. Numbers unique to each branch are coloured according to morphological compartment; branch length is defined as SNV number, and is to scale as shown. (C) cCGH analysis of the invasive components identified DNA copy number alterations common to both lesions, suggesting that they were derived from a common neoplastic clone. Chromosomal regions in blue/red were gained/deleted in the relevant lesion.

Interestingly, among cases with both LCIS and ILC, the staining pattern for E‐cadherin was concordant in these components in eight of 10 cases. In all cases with DCIS in the absence of LCIS, the invasive lobular component showed aberrant E‐cadherin staining. As exemplified by MDL4 in Figure 2A, some cases had a ‘transition zone’ in which it appeared that there was a direct morphological transition from ductal to lobular phenotypes coinciding with a progression from linear ductal E‐cadherin staining to an aberrant appearance as fragmented membranous and/or punctate, cytoplasmic staining in lobular regions.

### Morphologically and spatially distinct components of MDLs can arise from a common precursor

To explore clonal relationships between MDL components, morphologically distinct regions of DCIS, LCIS and invasive tumours with a ductal or lobular growth pattern were laser capture‐dissected or needle‐dissected from seven cases (supplementary material, Table S1), and analysed for DNA copy number alterations by cCGH (MDL1–MDL4, *n* = 4) or nucleotide variation by exome sequencing (MDL4–MDL7, *n* = 4). All samples were from FFPE diagnostic blocks, because appreciating the detailed morphology/cytology of individual components and understanding the topographical relationships between lesions within a case were critical to understanding the clonal nature of the molecular data obtained. Fresh frozen material may provide better‐quality input DNA for these types of analysis, but lack the morphological detail provided by FFPE samples.

Detailed morphological annotation and molecular data for cases analysed by cCGH are shown in Figure 2 and supplementary material, Figures [Link], [Link]. Illustrating the complexity of some cases, MDL1 contained DCIS, LCIS and both invasive ductal and lobular components within the same tissue block. DCIS and LCIS were sometimes admixed within the same duct, but showed different cytological features and staining for E‐cadherin and β‐catenin (supplementary material, Figure S1). We found that all morphological components analysed within the same case shared a considerable number of chromosomal aberrations (more than five events), suggesting they derived from a common ancestor before undergoing genotypic and phenotypic (morphological) change (Figure 2; supplementary material, Figures [Link], [Link] and Table S2).

**Figure 3:**
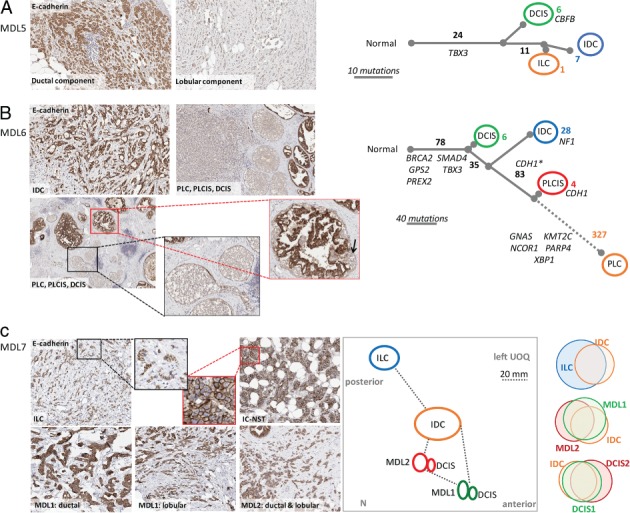
Genomic evolution of morphologically distinct components of MDL. (A) MDL5, showing E‐cadherin immunohistochemistry and the proposed molecular evolutionary tree. DCIS, IDC and ILC components were sequenced, and showed a shared ancestry of 24 clonal mutations. (B) MDL6, showing E‐cadherin staining of various morphological components and the proposed molecular evolutionary tree. CDH1* indicates the CDH1 p.Gln610* mutation, which is present at a low frequency in the DCIS component. CDH1 annotated on the PLC variant refers to the p.Arg796fs deletion. Some additional variants were shared between certain components, but did not fit well with the broader evolutionary tree. For example, two additional variants were detected in IDC and PLC only; IDC and DCIS shared two variants; and, IDC, DCIS and PLCIS shared one variant. The branch length is to scale, excluding the dotted line. (C) MDL7, showing E‐cadherin staining of various morphological components. A topographical map of MDL7 lesions is also shown (N, nipple; UOQ, upper outer quadrant of the breast). Proportional Venn diagrams demonstrate similarities between the various components (synonymous and non‐synonymous SNVs). Specific variants in each component are detailed in supplementary material, Table S4.

To investigate further, we performed multiregion WES and phylogenetic assessment of multiple components from four cases [MDL4–MDL7; the average sequencing depth for tumour DNA was 137‐fold (range: 110–194); the matched normal DNA average was 162‐fold (range 96–227); supplementary material, Tables S3 and S4]. Two approaches were applied to orthogonally validate the exome sequencing. First, nine variants were examined by iPlex genotyping of 15 samples; seven of nine tested variants were validated under these conditions (supplementary material, Table S5). Second, a trio of samples from MDL4 were subjected to RNA sequencing (RNAseq); FFPE‐derived ILC‐enriched and IDC‐enriched regions, and a fresh frozen sample of ILC. The RNAseq data validated 44.1% (56/127) of mutations in the IDC sample, and 40.9% (67/164) of mutations in the ILC sample (supplementary material, Table S6). These proportions are expected, as not all of the genes or mutated alleles will be expressed 18. A total of 261 single‐nucleotide variants (SNVs) and small insertion‐deletions were identified (supplementary material, Tables S3 and S4); 142 SNVs were validated by iPlex or RNAseq (supplementary material, Tables S5 and S6). The proportion of shared variants within a case was considerable, clearly indicating clonal relatedness between all components within individual tumours. Unique alterations were also identified, permitting assessment of the genetic distance between different components. Phylogenetic relationships between different components in four independent cases were investigated using a deductive reasoning approach, supported by PyClone analysis 19 (supplementary material, Data S1) and results are discussed below (Figures 2 and 3).

Invasive ductal and lobular components of MDL4 were analysed with cCGH and by exome sequencing (Figure 2B, C). Both invasive components were genetically complex, with considerable numbers of gains and losses, and SNVs. The two components shared 62% of gains and losses, and 72.7% of SNVs (Figure 2; supplementary material, Tables S3 and S4), with shared SNVs affecting the cancer driver genes *TP53* [p.Tyr220Cys, moderate effect (SNPeff), common mutation with 354 reported in COSMIC], *ESR1* (p.Ser154Leu, moderate effect, none recorded in COSMIC), and *TBX3* (p.Glu402_Pro403insGluGlu moderate effect, none recorded in COSMIC). Strong evidence supports a common ancestral relationship between lesions and likely progression of DCIS to IDC to ILC (in the absence of LCIS). The transition from a ductal to a lobular growth pattern was underpinned by a change in E‐cadherin complex function, as demonstrated by disparate immunohistochemical staining between the components (Figure 2).

In MDL5, DCIS, IDC and ILC were sequenced and, although lesions showed a relatively low mutational load, >75% of SNVs identified were shared among lesions, including in the driver gene *TBX3* (splice site alteration; predicted high effect, none recorded in COSMIC). Although *CDH1* was not mutated, the lobular component was negative for E‐cadherin, β‐catenin and p120 catenin by IHC (Figure 3A; supplementary material, Figure S4).

In MDL6, DCIS, IDC, pleomorphic LCIS and pleomorphic invasive lobular carcinoma (PLC) growth patterns were identified. The ductal components were E‐cadherin‐positive, whereas the pleomorphic lobular components were negative, and DCIS and pleomorphic lobular carcinoma *in situ* (PLCIS) were admixed in some ducts (Figure 3B; supplementary material, Figure S5). Seventy‐eight variants were shared by all four components, including mutations in several driver genes (e.g. *BRCA2*, p.Lys936fs, high effect; *SMAD4*, p.Arg361His, moderate effect, 76 events reported in COSMIC; *TBX3*, p.Pro42fs, high effect). The SNV load was higher than in other cases, and the PLC component was associated with the highest proportion of unique variants (53%). The pathway to invasion from the PLCIS branch featured 327 unique variants, including changes in several driver genes (e.g. *NCOR1*, p.Ser1117*, high effect, none recorded in COSMIC; *PARP4*, p.Gln147Glu, moderate effect, alternative missense mutations recorded in COSMIC). A *CDH1* nonsense mutation (p.Gln610*, high effect, reported missense mutations at that nucleotide) was identified in the DCIS (mutant allele frequency of 16%), PLCIS (85%) and PLC (75%) components; this variant was also present in three of 257 reads (1%) in the IDC. We restricted the *CDH1* mutation annotation to the pleomorphic branch in the tree after considering both the high frequency of the mutations in the pleomorphic regions and the admixture of cells. It is expected that the presence of this variant in ductal components results from contaminating ‘lobular’ cells, particularly given the admixture of DCIS and PLCIS within the same ducts, which is only evident on E‐cadherin‐stained sections (as indicated by the arrow in Figure 3B). Interestingly, PLCIS also harboured a 4‐bp deletion in *CDH1* (p.Arg796fs, high effect at 10% frequency, not recorded in COSMIC; supplementary material, Table S4).

MDL7 presented a vastly more complex situation, with the diagnostic pathology report showing a clear topography of four coincident tumours: ILC, IDC, and two MDLs (MDL1 and MDL2) (Figure 3C; supplementary material, Figure S6). Despite the morphological differences and physical distance between tumours, exome sequencing demonstrated that the most physically distant lesion contained the most unique variants (ILC, n = 3), as did MDL1, with which the ILC also shared several alterations (Figure 3C; supplementary material, Table S4). Indeed, one is reminded of the original ‘sick lobe’ studies; despite the physical distance, the shared changes indicate a clear ancestral history 20. Notably, no breast cancer driver genes were noted to be altered in this case.

## Discussion

This study investigated the clinical, pathological and molecular features of a large cohort of MDLs, and corroborates that MDLs constitute a distinct morphological entity 3, 4 with both unique biological features and some similarities with pure IDCs and ILCs. There is now strong evidence independently confirming that MDLs: are mostly grade 2 (58%); are more frequently associated with DCIS than with LCIS; are frequently lymph node‐positive (68.3%); are more frequently ER‐positive than IDCs; and, from a clinical point of view, have a prognosis that may lie somewhere between that for IDCs and that for ILCs 3, 4.

Here, we wished to gain a better understanding of the underlying aetiology of mixed tumours. The current multistep model of progression considers that IDC arises from DCIS, whereas ILC arises from LCIS, via a low‐grade or high‐grade pathway of tumourigenesis 21. It has so far been unclear how MDLs would fit into this theory. Two observations are worthy of elaboration as important insights. First, MDLs are more frequently associated with DCIS than with LCIS. In our series, DCIS was present in 87.9% of cases (60.3% as pure DCIS; 27.6% as coincident DCIS and LCIS), similar to what was reported by Rakha *et al* 3 (89%), whereas just 12.1% of cases had pure LCIS. It is possible that LCIS was present in more cases but was just not detected, or was completely transformed into the invasive lobular component. However, the differing distributions of DCIS and LCIS imply that the invasive lobular component of an MDL may be the result of evolution from a tumour clone of ductal origin in cases without LCIS.

Second, the expression status of E‐cadherin and the associated complex protein β‐catenin in the lobular components of MDLs was different from that seen in the ductal components of MDLs, from that seen in IDCs, and, importantly, from that typically seen in ILCs 22. In the vast majority of ductal lesions, E‐cadherin is expressed and linearly distributed on the cell membrane. ILCs show complete loss of E‐cadherin and β‐catenin expression in 90% of cases, and expression of these proteins but aberrant localization in the remaining 10% of cases 23. E‐cadherin expression in MDLs has previously been characterized as either positive (ductal‐like) or negative (lobular‐like 22). Here, we separately recorded the expression patterns in ductal and lobular areas, and, in most cases, the ductal components showed normal membranous staining. In 70.6% of cases, E‐cadherin and β‐catenin were expressed but were distributed in an aberrant localization, either as fragmented membrane staining, as diffuse cytoplasmic accumulation, or as strong focal perinuclear accumulation. This observation suggests that dysfunction of cell–cell adhesion may underpin the lobular growth pattern in MDLs, as it does in ILCs, although the mechanism of disruption is likely to be different between ILCs (*CDH1* mutation, loss, or silencing 16) and MDLs.

The primary objective of the molecular approach used was therefore to determine whether an invasive lobular growth pattern can evolve from a ductal origin, and, if so, whether we can uncover the molecular mechanism underpinning this progression. Although frozen samples are best for molecular analyses, we studied FFPE diagnostic cases to provide morphological clarity in the topographical relationships between the lesions analysed. Using both cCGH and exome sequencing we demonstrated that all morphological components within a given case shared a significant number of mutations (copy number alterations or SNVs), unequivocally demonstrating that all lesions within a case shared a common ancestry. Historically, these molecular approaches have demonstrated similar clonal relationships between columnar cell lesions and DCIS, between DCIS and associated IDC, between DCIS and coincident LCIS, and between LCIS and coincident ILC 10, 24, but, as far as we are aware, not between the different invasive components of MDL. This supports findings obtained with methylation analysis of the *HUMARA* gene to demonstrate that these components were related 25.

**Figure 4:**
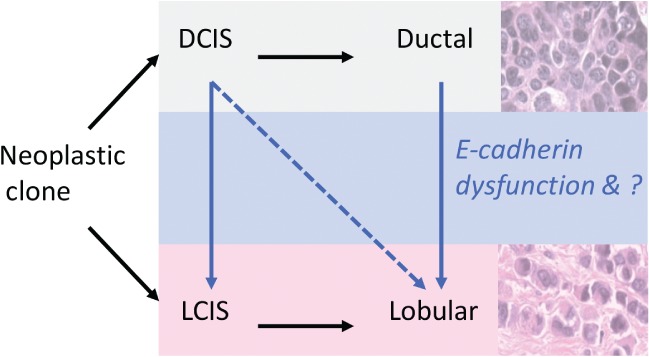
Hypothetical model for the evolution of different morphological components in MDL.

Historically, E‐cadherin expression in MDLs has been characterized as either positive (ductal‐like) or negative (lobular‐like 22); however, we showed previously the presence of a detectable but aberrant E‐cadherin staining pattern in some lobular‐like tumours 23. This implies that the evolution of the invasive lobular component may differ according to the presence of LCIS; there may be more than one pathway to the development of a lobular phenotype. These hypothetical concepts regarding the evolution of different morphological components of MDLs are summarized in Figure 4.

The largest molecular study of MDLs to date was performed by Ciriello *et al* 15, involving multi‐omic profiling of 88 MDLs as part of a larger breast cohort. The main focus of the analysis was to show whether these tumours could be classified as either ILC‐like or IDC‐like, based on the mRNA and somatic mutations; the conclusion was that MDLs do not represent a molecularly distinct entity, but are closely related to either IDC or ILC. Notably, the ILC‐like subgroup constituted ∼25% of MDL cases, and showed a higher rate of *CDH1* mutations and lower expression of E‐cadherin mRNA and protein. These findings could be interpreted in two ways. First, they may not have any biological relevance, owing to a critical lack of morphological annotation of the samples, and hence may simply reflect the proportions of the ductal or lobular components that underwent profiling. Alternatively, the authors may have highlighted, with their broad analysis, that ILC‐like cases represent those that may arise via LCIS and the loss of E‐cadherin by gene mutation, whereas the IDC‐like cases represent those arising via the ductal pathway, with no LCIS component and aberrant E‐cadherin expression for unknown reasons.

The molecular analyses identified a number of defining genomic alterations; in particular, the exome sequencing identified several key cancer driver mutations, particularly in the common ancestral evolutionary arm of each case. For example, we identified a frameshift deletion in *BRCA2* (c.2806_2809delAAAC) in the evolution of MDL6 that involved a pleomorphic lobular component; a relationship between *BRCA2* variants and pleomorphic lobular breast cancer has previously been suggested 26. Large‐scale genomics consortia have previously identified a number of breast cancer driver genes, including *TBX3* 27; indeed, *TBX3* mutations account for ulnar–mammary syndrome 28, and, in breast cancer, appear to result in the loss of transcriptional repressor function 29. Intriguingly, *TBX3* alterations are enriched in ILCs as compared with IDCs (9.5% versus 1.6% 18), and we, too, report genetic alterations in *TBX3* in three of four WES cases, all of which were early events. Functionally, TBX3 has been shown to repress E‐cadherin expression in melanoma 30, and it is a downstream target of the Wnt–β‐catenin pathway 31. The interplay between *TBX3* mutations and the E‐cadherin adhesion complex in MDLs is a fascinating area for future research.

This study demonstrates that the different morphological components present within an MDL tumour are clonally related and not the result of a collision of multiple independent tumours, thus supporting the idea that MDL tumours represent a distinct clinical and biological entity. We show that, in some cases, the divergence of the morphological components may occur early during tumour evolution (both DCIS and LCIS are present) or later during tumour progression (only DCIS detectable). The cases with late‐occurring divergence may arise via a ductal‐like pathway of progression, and these data emphasize the possibility that a lobular‐like phenotype can arise via a modified ductal pathway. As previously detailed 3, the clinical conundrum surrounding a mixed tumour is that the good prognostic features of one component may have no prognostic value in the presence of a component with more aggressive features, regardless of which is the most conspicuous component. Clinically, there is little value in broadly categorizing MDLs into either lobular‐like or ductal‐like, and a more detailed assessment is required. Indeed, we have shown that the morphologically disparate regions harbour mutations in several breast cancer driver genes, which may predict targeted therapeutic options in the future. The evaluation of the true molecular profile of breast tumours is greatly influenced by tumour cellularity, and, critically in terms of discovery approaches, known proportions of morphologically distinct clones. The molecular analysis presented here highlights the genomic differences and similarities between the morphological regions of the MDLs, and contributes to our broader understanding of the genetic picture of breast cancer.

## Author contributions statement

AMR designed and performed experiments, analysed data, and wrote the paper. JRK performed experiments, analysed data, and wrote the paper. KN performed experiments, analysed data, and wrote the paper. FN, SK, JJ, ACV, AMP, XQC, and LER performed experiments. LDS, LM, and MC performed pathology review. JMS and JMB designed experiments and analysed data. EE and AP provided funding and tissue resources. GCT provided funding and designed experiments. DC provided tissue resources. NW and JP analysed data and wrote the paper. SRL provided funding and tissue resources, supervised the pathology review, and conceived the study. PTS scored the IHC, analysed data, wrote the paper, provided resources, and conceived the study.

**SUPPLEMENTARY MATERIAL ONLINE**

**Supplementary materials and methods**

**Supplementary figure legends**

**Data S1.** Pyclone analysis

**Figure S1.** Detailed morphology and additional IHC of MDL1

**Figure S2.** Detailed morphology and additional IHC of MDL2

**Figure S3.** Detailed morphology and additional IHC of MDL3

**Figure S4.** Detailed morphology and additional IHC of MDL5

**Figure S5.** Detailed morphology and additional IHC of MDL6

**Figure S6.** Detailed morphology and additional IHC of MDL7

**Table S1.** Pathology information and experimental summary of MDL cases

**Table S2.** Summary of chromosomal copy number alterations

**Table S3.** Exome data

**Table S4.** Curated nucleotide variant list

**Table S5.** Validation of alterations by iPlex, and list of iPlex primers

**Table S6.** MDL4 RNASeq data

## Supporting information


**Supplementary materials and methods**

**Supplementary figure legends**

**Data S1.** Pyclone analysis

**Figure S1. Detailed morphology and additional IHC of MDL1.** (A) Case MDL1 contained LCIS and DCIS, and invasive components with both ductal [D] and lobular [L] growth patterns. H&E stained sections show the admixed relationship between these morphological components. DCIS and invasive ductal components were E‐cadherin and b‐catenin positive. LCIS and invasive lobular components were E‐cadherin weak/aberrant and β‐catenin negative. (N: Normal lobule). (B) cCGH analysis of the four components of MDL1 identified DNA copy number alterations common to all lesions, suggesting they were derived from a common neoplastic clone. Chromosomal regions in blue/red were gained/deleted in the relevant lesion, respectively. Amplification of 11q suggested CCND1 may be amplified and overexpressed; IHC (in A) demonstrated all cells of all components were strongly positive for Cyclin D1 protein.

**Figure S2. Detailed morphology and additional IHC of MDL2.** MDL2 contained DCIS and LCIS (not shown) and invasive components with both ductal [D] and lobular [L] growth patterns. Stained sections show the admixed relationship between tumour nests and single cells and single cell files. The three different morphological components were membrane positive for E‐cadherin, β‐catenin and P120‐catenin; negative for vimentin and N‐cadherin (not shown) and were 3+ positive for HER2. All images are at 20x. cCGH analysis of the invasive components identified a greater number of DNA copy number alterations common to both lesions, than the number unique to each morphology. Chromosomal regions in blue/red were gained/deleted in the relevant lesion.

**Figure S3. Detailed morphology and additional IHC of MDL3.** MDL3 contained DCIS (not shown) and invasive components with both ductal [D] and lobular [L] growth patterns. H&E stained section show the admixed relationship between tumour nests and single cells. The DCIS and invasive ductal components were E‐cadherin, β‐catenin and P120‐catenin positive. The single cells of the invasive lobular component showed cytoplasmic reactivity for E‐cadherin and P120‐catenin and weak/negative staining for β‐catenin. All tumour cells were negative for vimentin and N‐cadherin and were 3+ positive for HER2. All immunohistochemistry images are at 20x. cCGH analysis of the invasive components of MDL3 identified DNA copy number alterations common to both lesions suggesting they were derived from a common neoplastic clone. Chromosomal regions in blue/red were gained/deleted in the relevant lesion.

**Figure S4. Detailed morphology and additional IHC of MDL5.** IDC and ILC components stained for β‐catenin and p120‐catenin.

**Figure S5. Detailed morphology and additional IHC of MDL6** Additional immunohistochemical staining of β‐catenin and p120‐catenin for different morphological components of case.

**Figure S6. Detailed morphology and additional IHC of MDL7.** Additional immunohistochemical staining of β‐catenin and p120‐catenin for different morphological components of case.

**Table S1.** Pathology information and experimental summary of MDL cases
**Table S2.** Summary of chromosomal copy number alterations
**Table S3.** Exome data
**Table S4.** Curated nucleotide variant list
**Table S5.** Validation of alterations by iPlex, and list of iPlex primers
**Table S6.** MDL4 RNASeq data
