# Supplementary material for: Mixed ductal‐lobular carcinomas: evidence for progression from ductal to lobular morphology
Source: J Pathol. 2018 Mar 9;244(4):460–8. doi: 10.1002/path.5040 (PMC5873281; doi:10.1002/path.5040)
Supplement: Supplementary file 1 — Supplementary materials and methods [file PATH-244-460-s003.docx]

**Supplementary materials and methods**

Reference numbers refer to the main text list

*Immunohistochemistry*

Immunohistochemistry (IHC) was performed on whole tissue sections under the following conditions (all antibodies were incubated at room temperature for 1 h unless otherwise specified): E-cadherin, 1:100 at 4 °C for 16 h, clone HECD1 (Invitrogen, Life Technologies Australia Pty Ltd. Mulgrave, Australia); β-catenin, 1:100, clone 17C2 (Novocastra, Life Technologies Australia Pty Ltd.); p120-catenin, 1:200, clone 98/pp120 (BD Transduction labs, North Ryde, Australia); N-cadherin, 1:150, clone 3B9 (Invitrogen); Snail, 1:40 at 4 °C for 16 h, clone SN9H2 (Cell Signaling, Genesearch Pty. Ltd. Arundel, Australia); Vimentin, 1:400, clone V9 (Dako Australia Pty. Ltd., Campbellfield, Australia). All antibodies required citrate (pH 6.0) antigen retrieval at 125 °C for 5 min. The MACH 1 Universal HRP-Polymer kit (Biocare Medical, Concord, USA) was used for detection. The expression status of ER, PR and HER2 for each case was retrieved from pathology reports.

Tumour microdissection and DNA extraction

Tumour-rich areas were identified and five 10 μm tissue sections were cut onto either plain slides (for needle macrodissection) or polyethylene naphthalate membrane-coated slides (for laser capture microdissection (LCM); Leica Microsystems, North Ryde, Australia). The tissue was deparaffinised, stained with Nuclear Fast Red and rehydrated through a graded ethanol series. Tissue samples were microdissected using a Leica LMD 7000 LCM microscope and areas of interest were collected into tubes containing ATL buffer (Qiagen, Chadstone, Australia) then digested overnight with 10 μl of proteinase K (Invitrogen) at 55 °C with shaking. Macrodissection was performed under a stereomicroscope using a fine needle and tissue was digested in ATL buffer (Qiagen) with proteinase K in a shaking incubator at 55 °C for 3 d, with proteinase K replenished daily to ensure complete tissue digestion. For both dissection methods, DNA was extracted using a Qiagen QIAmp DNA Micro Kit following the manufacturer’s instructions. Normal lymph nodes were used as a source of normal genomic DNA.

*Chromosomal comparative genomic hybridization*

Cases that underwent CGH and exome sequencing are summarised in supplementary material, Table S1. Chromosomal CGH was performed and analysed as described previously [10] and data summarised in supplementary material, Table S2.

*Whole exome Sequencing*

WES was performed at Queensland Centre for Medical Genomics using the Illumina Nextera Rapid Capture Exome Kit (Illumina, Scoresby, Australia) following the manufacturer’s instructions. Sequence data was adapter trimmed using cutadapt (version 1.8.1), aligned to the GRCh37 assembly using BWA-MEM (v 0.7.12-r1039) and converted to coordinate sorted lane aligned read group binary alignment mapping files with samtools 1.1 (using htslib 1.1). The aligned read groups were merged by sample. Coverage was estimated using qCoverage (<http://sourceforge.net/p/adamajava/wiki/qCoverage/>) per sample (supplementary material, Table S1). Point mutations were called by The Genome Analysis Toolkit (GATK) [32] and qSNP [33]. Prior to qSNP analysis sequence reads were filtered to include only those with an alignment length >34 or second in a pair and mapped as a proper pair; less than 3 mismatches to the reference genome and not a PCR duplicate. High confidence variants were those called by both GATK and qSNP with position covered with 8 or more reads in the tumour, 12 or more in the normal and with a variant read of >4. Variants were annotated with population frequencies from dbSNP (v141), and gene consequence using SnpEff4.0e (build 2014-09-13), data is in supplementary material, Tables S3, S4. When multiple tumour samples were available from the same patient, variants were classified as unique to a sample or shared. For clonal evolution, we manually reviewed the variant allele frequencies, and included variants from additional samples within the same patient, which had not been called confidently as a mutation, but which occurred at a low frequency. Variants re-included had to have a read depth in the normal sample of at least approximately equal to that of the tumour sample, in an effort to minimize error.

*Sequence validation*

Genotyping of variants was carried out as part of a multiplexed assay using the MALDI TOF spectroscopy MassARRAY platform (Agena, Herston, Australia) and iPLEX GOLD chemistry (Agena). Genomic DNA was used as template (10 ng) with Qiagen HotStarTaq. Shrimp alkaline phosphatase and primer extension steps were carried out according to Agena protocols. Primers were obtained from Integrated DNA Technologies (Ohio USA). Assays were designed with MassARRAY Assay Design version3.1. Raw genotype data were visualised and processed with MassARRAY Typer software version3.4 [34]. Primers and data are provided in supplementary material, Table S5. The selected variants were tested in each of 15 samples.

*RNASeq*

Macrodissected MDL4 ductal and lobular and normal regions (FFPE), and fresh frozen tissue of the lobular component were extracted using Qiagen RNeasy FFPE and RNeasy kits, respectively, and prepared for sequencing using the TruSeq Stranded Total RNA kit (Illumina). Ribosomal depletion and library construction was performed on 500 ng total RNA according to the manufacturer's protocol. The libraries were quality checked and quantified using a Bioanalyser High Sensitivity DNA kit (Agilent). All the RNA libraries were indexed, allowing multiplexed sequencing. Prior to on-board cluster generation (average density of ~910 K/mm^2^) and sequencing, the pooled library was denatured using 0.1 M NaOH and diluted to 7.5 pM. Libraries were sequenced as 100 bp paired-end reads using an Illumina HiSeq 2000. A pileup up approach was used to validate mutations, whereby the number of reads showing the reference genome base and variant base were counted at each mutation position. Data is provided in supplementary material, Table S6.

*Phylogenetic Analysis*

Phylogenetic analysis was based on identifying which chromosomal aberrations and single nucleotide variants (SNVs) were unique to a particular morphological component (‘unique’) or present in multiple components in the same tumour (‘shared’). Some unique SNVs were reclassified as shared if they were identified upon manual review (low-frequency SNVs, where total read depth in the normal sample was approximately equal to the tumour sample, and the variant read proportion was ≥3%). Simplified evolutionary trees were constructed using a deductive reasoning approach, wherein relative branch lengths indicate the number of SNVs in each branch, and the proportions of private and shared alterations were used to calculate evolutionary branch positions (as per [35]). Venn diagrams were created with Venny ([36]; https://www.stefanjol.nl/venny).

*Statistical analyses*

Statistical analyses were performed using GraphPad Prism v6.0. Contingency table associations were assessed for statistical significance using a Chi square analysis, however if sample sizes were insufficient, a Fisher’s Exact test was performed. P values are as annotated.
