# Supplementary material for: Mixed ductal‐lobular carcinomas: evidence for progression from ductal to lobular morphology
Source: J Pathol. 2018 Mar 9;244(4):460–8. doi: 10.1002/path.5040 (PMC5873281; doi:10.1002/path.5040)
Supplement: Supplementary file 3 — Data S1. Pyclone analysis [file PATH-244-460-s004.docx]

**Supplementary Data S1 : Pyclone analysis**

Reference numbers refer to the main text list

PyClone Analysis

We employed PyClone [19] to support our deductive reasoning approach to assessing the phylogenetic relationships between the morphological subclones. PyClone requires deep-sequence mutation data as well as allele specific copy number data. While adequate for calling mutations, our data was not of sufficient coverage to meet the PyClone input requirements. Furthermore, we used ExomeCNV [37] to generate the copy number information required by PyClone; again this is a work around, as SNP or whole genome-derived calls are optimal. When comparing our phylogenetic trees with those clones derived from the PyClone analysis, it is clear that the PyClone analysis supports our conclusions. The mutation burden of MDL7 was too low to run PyClone in a meaningful way.

Considering MDL4 (Figure A), the 6 graphs represent clonal variants, with clusters 10, 4, 8 and 9 showing ‘trunk’ clones with variants shared by both tumour components. Cellular prevalence (or frequency) of variant forms the Y axis, with each component of MDL4 on the X axis. Each graph is titled by cluster number, with the number of variants in parentheses. Clusters 1 and 3 represent the unique variants in the IDC and ILC components respectively, as one component shows zero prevalence.

In MDL5 (Figure B), clusters 1, 2, 5 and 6 represent the trunk mutations, while the IDC/ILC branch is made up of cluster 11. In MDL6 (Figure C) the trunk is populated by clusters 13, 7, 8 and 9. The MDL6 PLC/PLCIS branch is composed of clusters 2 and 4 and the unique clusters are 1 (PLCIS), 16 (PLC), 17 (IDC) and 18 (DCIS).


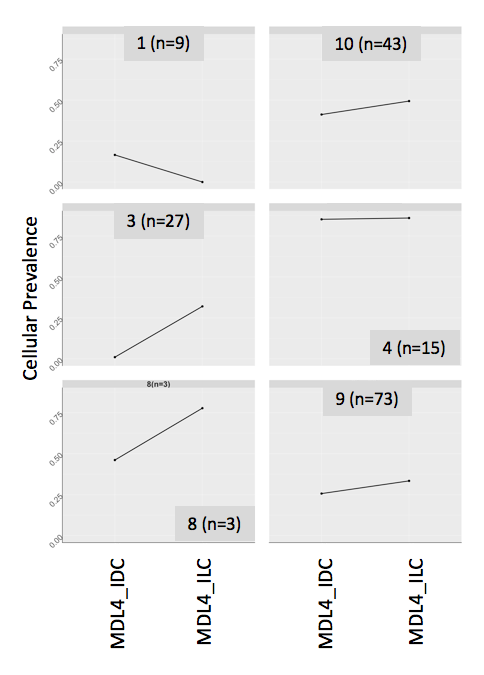


Figure A. PyClone clonal analysis of MDL4 identifies 6 subclone clusters. IDC and ILC share 4 clusters, and each has one unique.


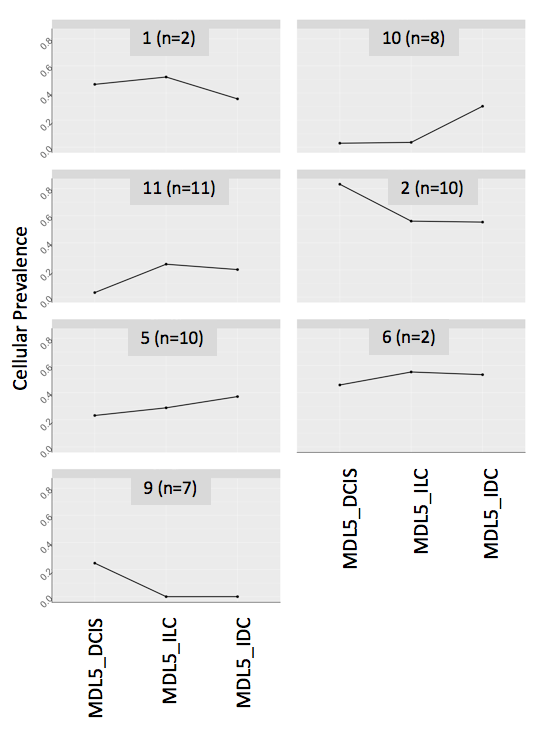


Figure B. PyClone clonal analysis of MDL5 identifies 7 subclone clusters.

*
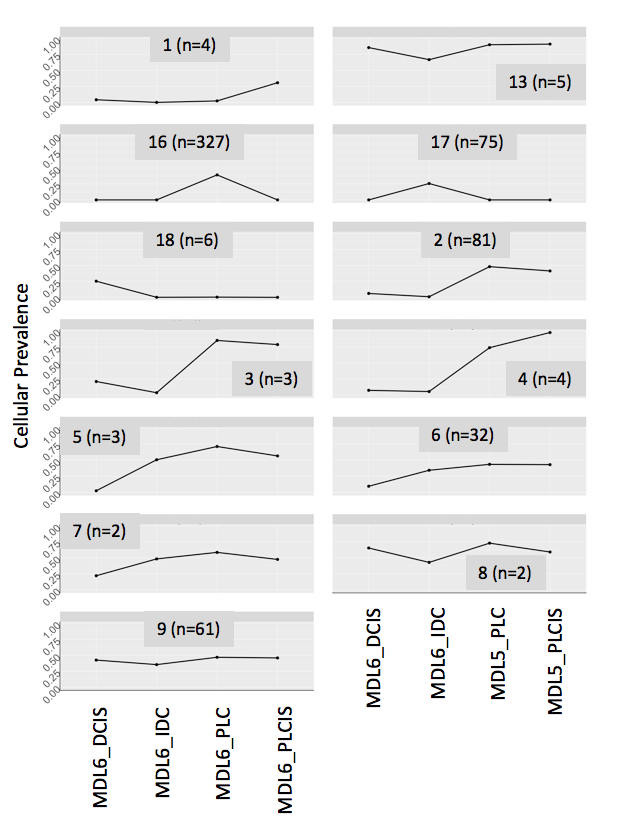
*Figure C. PyClone clonal analysis of MDL6 identifies 13 subclone clusters.
