# Supplementary figures and images for: Mixed ductal‐lobular carcinomas: evidence for progression from ductal to lobular morphology
Source: J Pathol. 2018 Mar 9;244(4):460–8. doi: 10.1002/path.5040 (PMC5873281; doi:10.1002/path.5040)

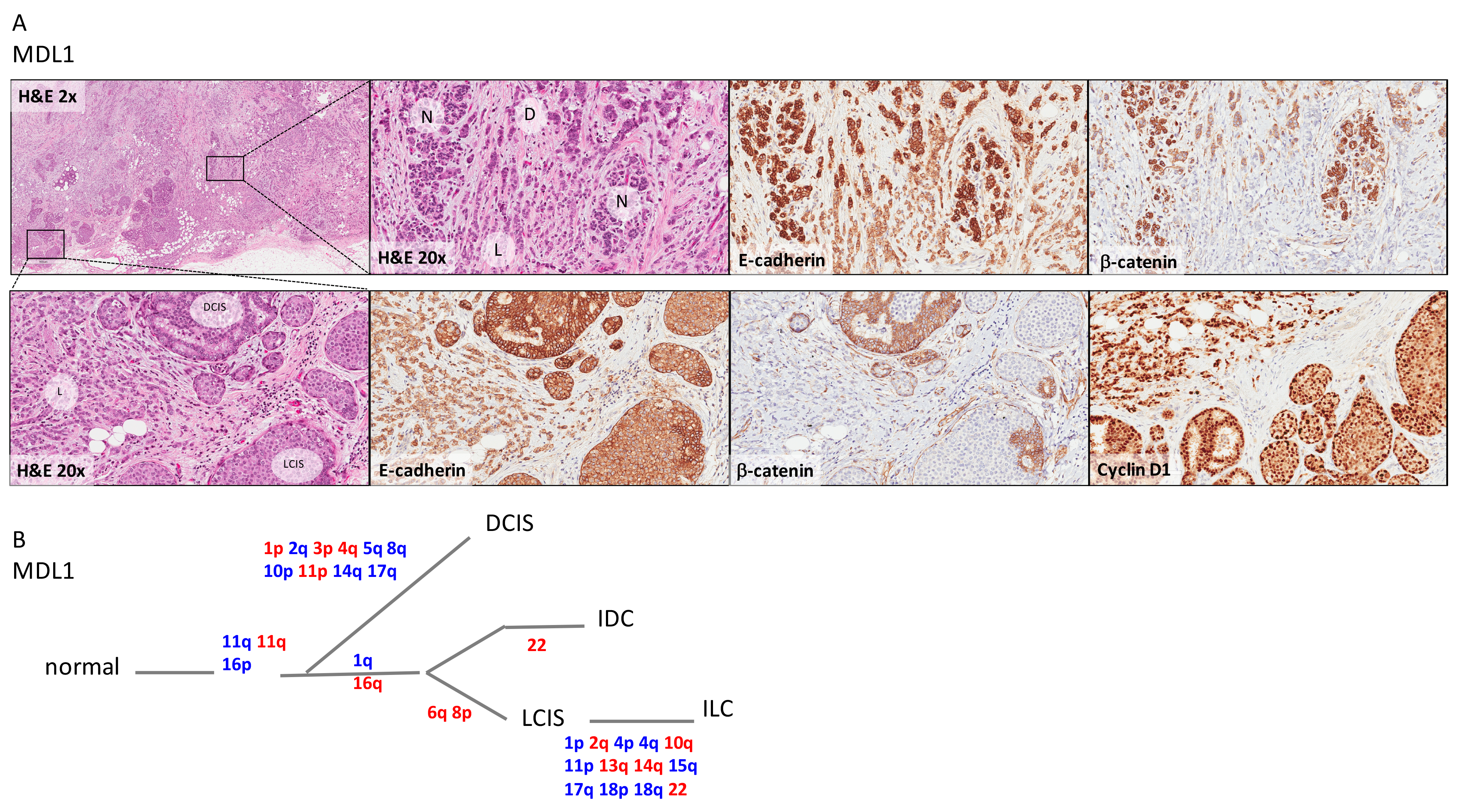

Supplement: Supplementary file 4 — Figure S1. Detailed morphology and additional IHC of MDL1. (A) Case MDL1 contained LCIS and DCIS, and invasive components with both ductal [D] and lobular [L] growth patterns. H&E stained sections show the admixed relationship between these morphological components. DCIS and invasive ductal components were E‐cadherin and b‐catenin positive. LCIS and invasive lobular components were E‐cadherin weak/aberrant and β‐catenin negative. (N: Normal lobule). (B) cCGH analysis of the four components of MDL1 identified DNA copy number alterations common to all lesions, suggesting they were derived from a common neoplastic clone. Chromosomal regions in blue/red were gained/deleted in the relevant lesion, respectively. Amplification of 11q suggested CCND1 may be amplified and overexpressed; IHC (in A) demonstrated all cells of all components were strongly positive for Cyclin D1 protein. [file PATH-244-460-s002.tif]

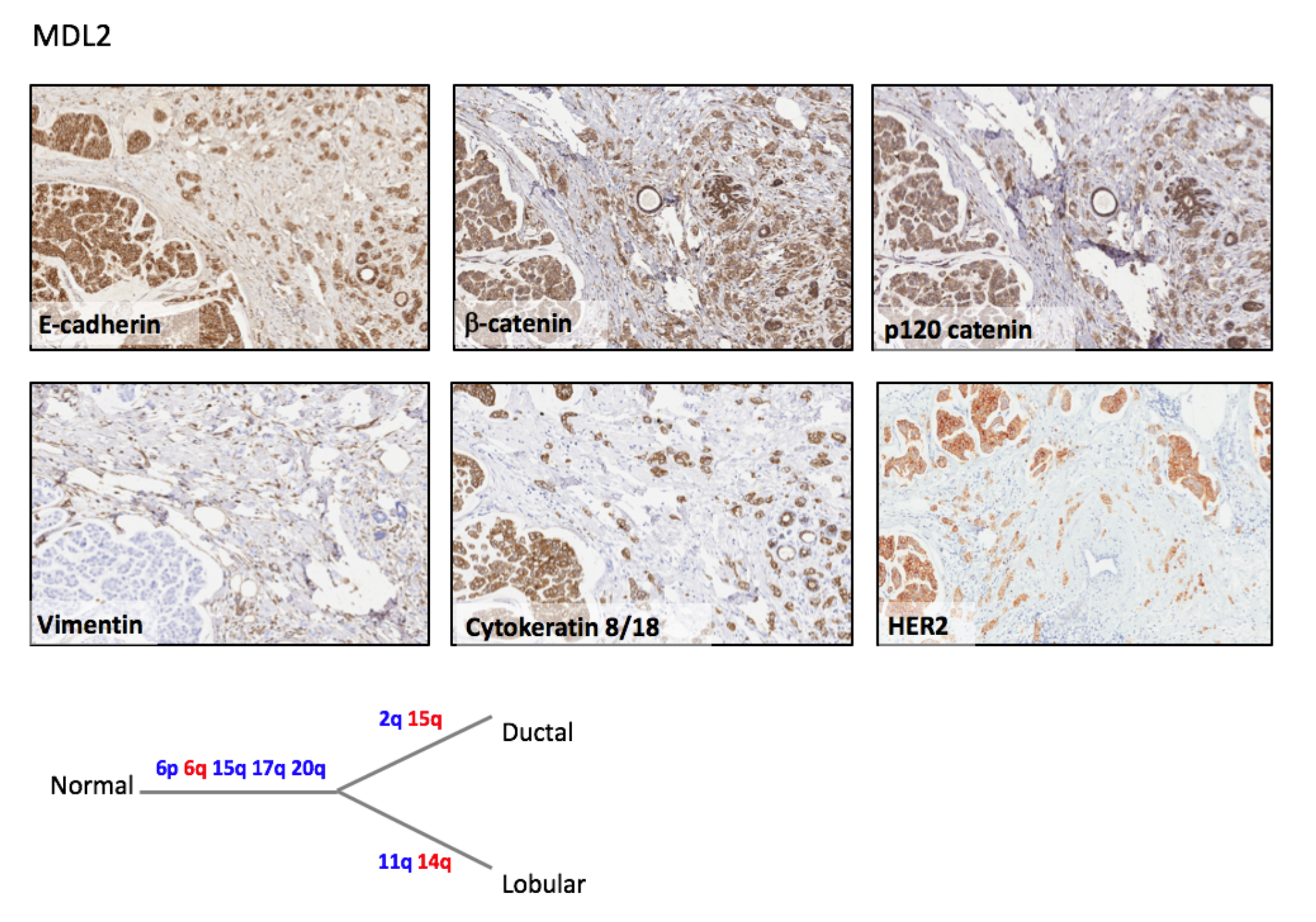

Supplement: Supplementary file 5 — Figure S2. Detailed morphology and additional IHC of MDL2. MDL2 contained DCIS and LCIS (not shown) and invasive components with both ductal [D] and lobular [L] growth patterns. Stained sections show the admixed relationship between tumour nests and single cells and single cell files. The three different morphological components were membrane positive for E‐cadherin, β‐catenin and P120‐catenin; negative for vimentin and N‐cadherin (not shown) and were 3+ positive for HER2. All images are at 20x. cCGH analysis of the invasive components identified a greater number of DNA copy number alterations common to both lesions, than the number unique to each morphology. Chromosomal regions in blue/red were gained/deleted in the relevant lesion. [file PATH-244-460-s001.tif]

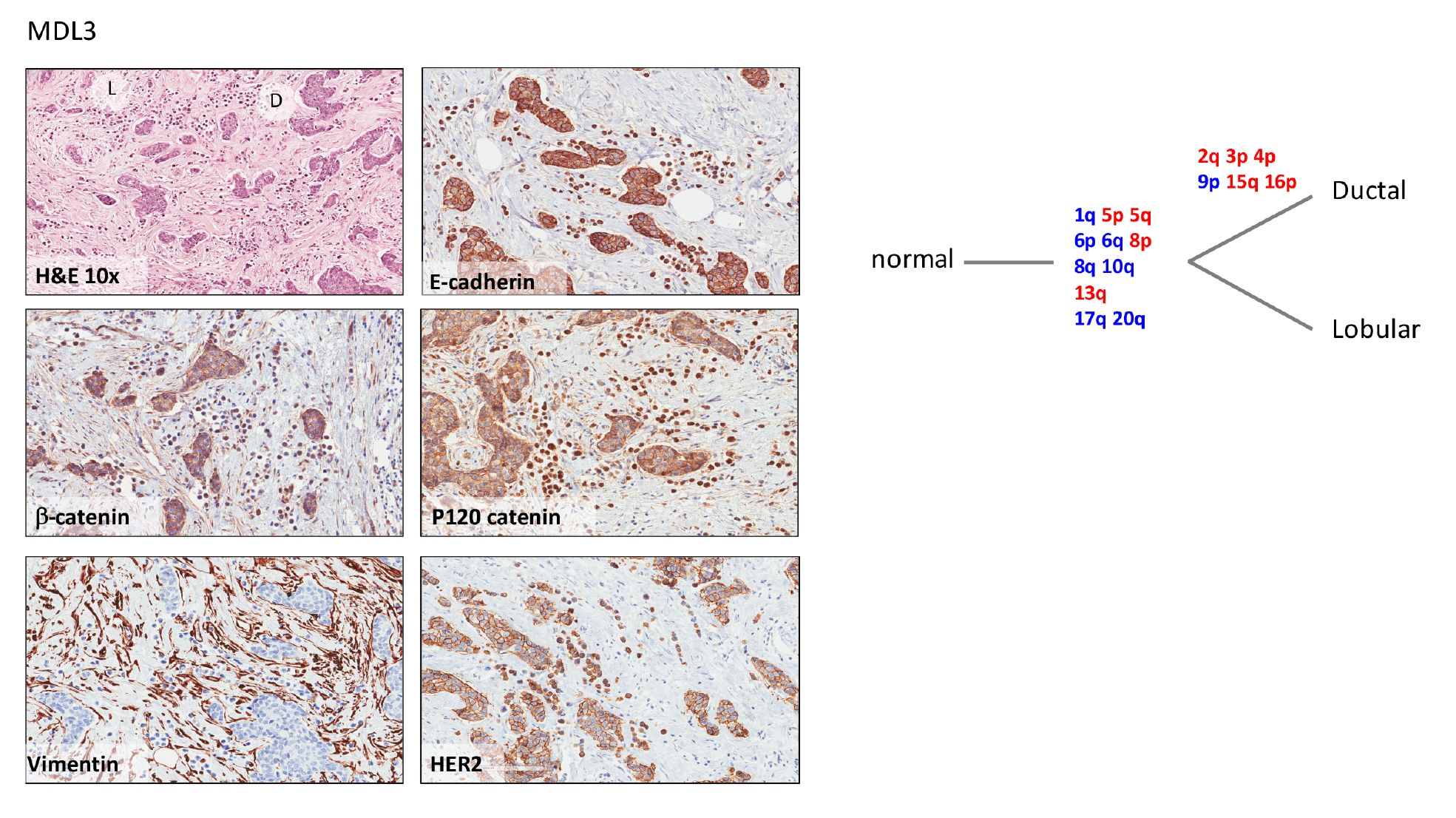

Supplement: Supplementary file 6 — Figure S3. Detailed morphology and additional IHC of MDL3. MDL3 contained DCIS (not shown) and invasive components with both ductal [D] and lobular [L] growth patterns. H&E stained section show the admixed relationship between tumour nests and single cells. The DCIS and invasive ductal components were E‐cadherin, β‐catenin and P120‐catenin positive. The single cells of the invasive lobular component showed cytoplasmic reactivity for E‐cadherin and P120‐catenin and weak/negative staining for β‐catenin. All tumour cells were negative for vimentin and N‐cadherin and were 3+ positive for HER2. All immunohistochemistry images are at 20x. cCGH analysis of the invasive components of MDL3 identified DNA copy number alterations common to both lesions suggesting they were derived from a common neoplastic clone. Chromosomal regions in blue/red were gained/deleted in the relevant lesion. [file PATH-244-460-s007.tif]

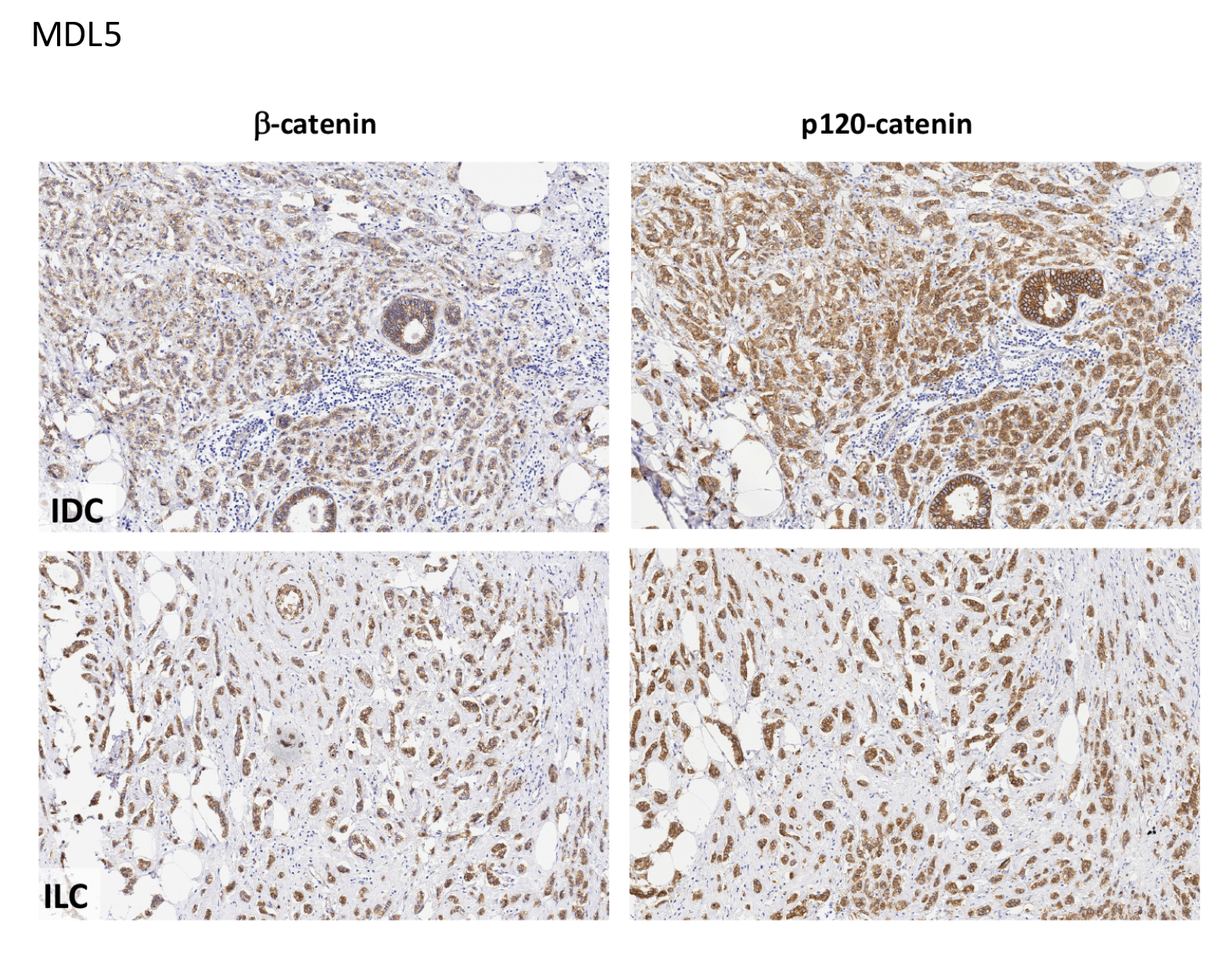

Supplement: Supplementary file 7 — Figure S4. Detailed morphology and additional IHC of MDL5. IDC and ILC components stained for β‐catenin and p120‐catenin. [file PATH-244-460-s009.tif]

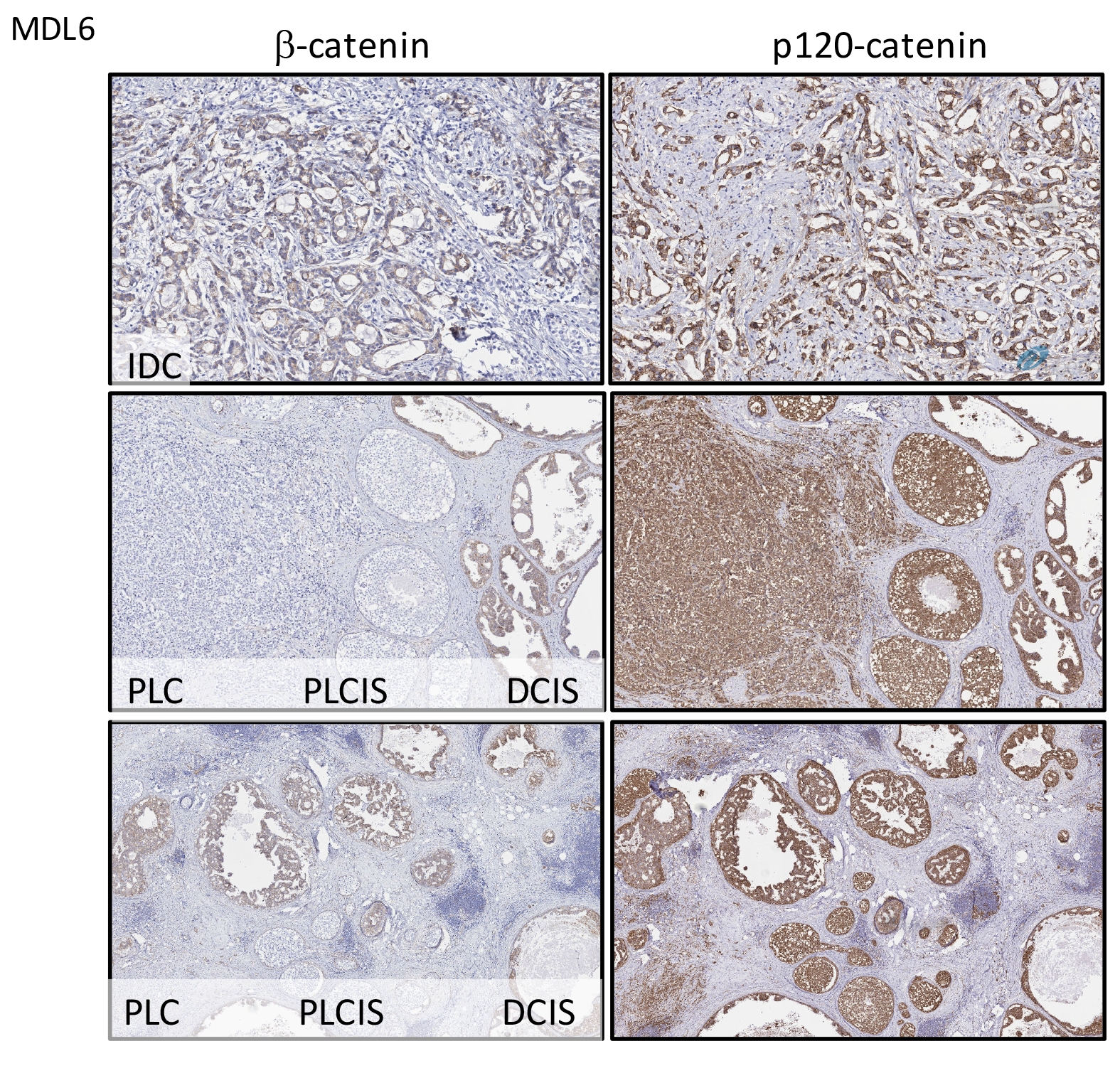

Supplement: Supplementary file 8 — Figure S5. Detailed morphology and additional IHC of MDL6 Additional immunohistochemical staining of β‐catenin and p120‐catenin for different morphological components of case. [file PATH-244-460-s006.tif]

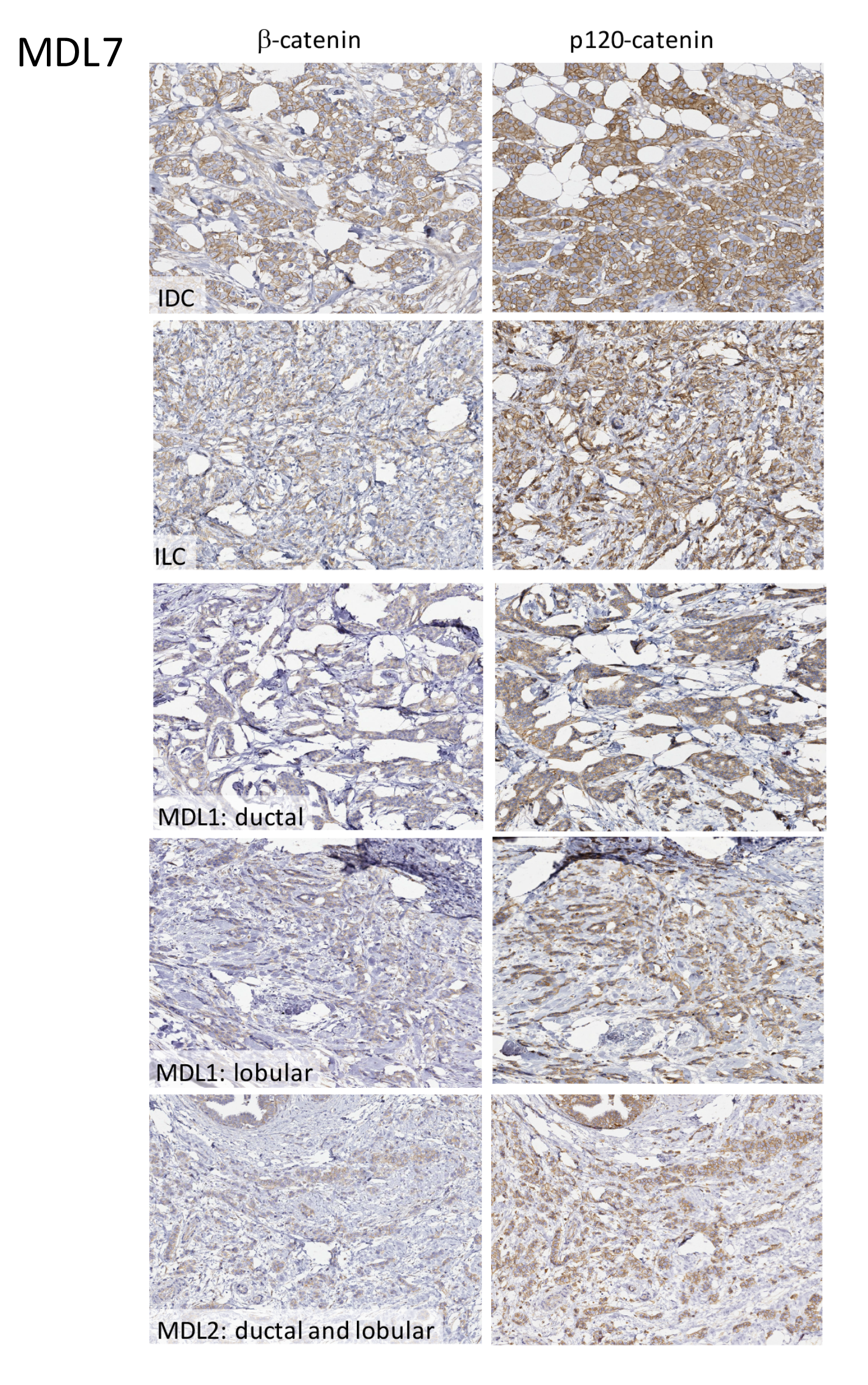

Supplement: Supplementary file 9 — Figure S6. Detailed morphology and additional IHC of MDL7. Additional immunohistochemical staining of β‐catenin and p120‐catenin for different morphological components of case. [file PATH-244-460-s010.tif]
